# Video-confidence: a qualitative exploration of videoconferencing for psychiatric emergencies

**DOI:** 10.1186/s12913-014-0544-y

**Published:** 2014-10-31

**Authors:** Marianne Vibeke Trondsen, Stein Roald Bolle, Geir Øyvind Stensland, Aksel Tjora

**Affiliations:** Norwegian Centre for Integrated Care and Telemedicine, University Hospital of North Norway, P.O. Box 35, N-9038 Tromsø, Norway; Division of Emergency Medical Services, University Hospital of North Norway, P.O. Box 45, N-9038 Tromsø, Norway; Department South, General Psychiatric Clinic, University Hospital of North Norway, P.O. Box 6124, N-9291 Tromsø, Norway; Department of Sociology and Political Science, Norwegian University of Science and Technology, N-7491 Trondheim, Norway

**Keywords:** Psychiatry, Emergency care, Videoconferencing, Tele-psychiatry, Confidence, Qualitative study

## Abstract

**Background:**

In psychiatric emergencies in rural areas the availability of psychiatrists are limited. Therefore, tele-psychiatry, via real-time videoconferencing (VC), has been developed to provide advanced consultative services to areas that lack psychiatrists. However, there is limited research on the use of VC for psychiatric emergencies. The University Hospital of North Norway has been the first hospital in Norway to implement this type of service by developing a new on-call system for psychiatric emergency practice through which psychiatrists are accessible by telephone and VC 24 hours a day for consultations with patients and nurses at three regional psychiatric centres. This study explores patients’, psychiatrists’ and nurses’ experiences of using VC for psychiatric emergencies, as well as how the technology influenced their confidence.

**Methods:**

In this study, we used a qualitative explorative research design. With a particular focus on users’ experiences of VC, we conducted 29 semi-structured interviews with patients, psychiatrists and nurses who had participated in a VC consultation in at least one psychiatric emergency.

**Results:**

Our findings show that access to the VC system increased the experience of confidence in challenging psychiatric emergencies in four ways: (1) by strengthening patient involvement during the psychiatric specialist’s assessment, (2) by reducing uncertainty, (3) by sharing responsibility for decisions and (4) by functioning as a safety net even when VC was not used.

**Conclusions:**

This study has demonstrated that an emergency psychiatric service delivered by VC may improve the confidence of psychiatrists, nurses and patients in challenging psychiatric emergencies. VC can serve as an effective tool for ensuring decentralised high-quality psychiatric services for emergency care.

## Background

De-institutionalisation, in the form of providing patient services through outpatient clinics and day treatment rather than hospital stays, is a strong and growing trend in healthcare services. In psychiatric hospitals, this process began in the 1960s and benefitted most psychiatric patients, although many were left without proper care [1]. The process of de-institutionalisation occurs today partly for economic reasons and partly because new treatment options are making it increasingly feasible. In addition, maintaining life in patients’ own communities is also believed to help them better manage their disease and their lives [1].

The Ministry of Health and Care Services in Norway aims to improve collaboration between the different levels of healthcare services, as well as to reduce unnecessary and compulsory admissions in acute psychiatric wards, by re-organising and de-institutionalising healthcare services [2]. However, such changes may be particularly challenging in rural areas, especially for psychiatric emergencies, where the availability of specialists is limited.

Tele-psychiatry, via real-time videoconferences (VC), is increasingly being used to provide advanced consultative services in areas that lack local access to psychiatrists [3]. The use of VC can improve this access by connecting psychiatrists to health personnel and patients from a great distance, and a broad range of mental health issues has been successfully handled using this technique. Studies have found that tele-psychiatric services used in non-urgent situations have increased patients’ access to therapy, increased patients’ satisfaction, saved time and reduced patients’ need for travel [3-10]. Young patients have stated that the use of VC communication in therapy alleviated their previous anxieties about consulting a psychiatrist [5]. In addition, via regular access to specialists through VC, health personnel experienced increased knowledge and improved confidence in assisting patients locally [3].

Both in psychiatric and somatic medicine, medical emergencies are characterised by complexity, uncertainty, lack of information, distances between patients and health personnel and decisions that may be retrospectively judged by others as suboptimal. The use of VC in these situations may therefore be thought of as challenging and as causing an unwanted shift of focus away from the patient and towards technology. At the same time, there is a need to develop new methods for providing proper emergency care for mentally ill patients in rural areas. The use of VC in psychiatric emergencies has been found to be safe, effective and satisfactory for both the patients and emergency health staff [11,12]. There is, however, limited research on the use of VC for psychiatric emergency consultations [11,12], and there is a need for increased knowledge of emergency tele-psychiatry in operation.

The aim of this article is to explore the experiences of patients, psychiatrists and nurses regarding the use of VC for consultations in psychiatric emergencies and how the technology influences their confidence. The article is based on a qualitative study of the implementation of the first Norwegian tele-psychiatric emergency service, which was established in 2011 by the University Hospital of North Norway [13].

## Methods

### Site of research

The Department South of the General Psychiatric Clinic at the University Hospital of North Norway consists of three regional psychiatric centres and an acute psychiatric hospital ward that is located in the county capital (Tromsø). The department serves 95,000 people living in the surrounding small cities, towns and rural communities. The regional centres are located in towns 2.5 to 4 hours away from the acute psychiatric ward in Tromsø, and each of the centres have a local psychiatric ward with 12 beds, an ambulatory psychiatric team and outpatient services. The regional centres are responsible for delivering psychiatric emergency services in their geographical areas. However, because of the geographical remoteness of the regional psychiatric centres, the department have not succeeded in recruiting enough psychiatrists to the regional centres to provide robust 24-hour emergency care. To overcome this challenge, in 2011, Department South implemented a new decentralised on-call system for psychiatric emergencies in collaboration with the Norwegian Centre for Integrated Care and Telemedicine [13]. VC studios were installed at the three regional psychiatric centres and in the offices or homes of six psychiatrists. The psychiatrists agreed to be accessible 24 hours a day, in a rotation scheme, for the regional centres’ ambulatory psychiatric teams and psychiatric wards and to be available to take part in direct patient consultations by VC in collaboration with nurses at the centres. The nurses are specially trained in psychiatric health care and are experienced in handling psychiatric crises. They generally work autonomously and only contact the psychiatrists when they need advice or help in evaluating the patient. The purpose of the new VC on-call service was to ensure that all patients had equal access to specialist assessments independent of their location.

### Research design

Because of the limited research evidence regarding the use of VC for psychiatric emergencies, a qualitative explorative research design was used in this study. With a focus on the users’ experiences of VC, we conducted semi-structured interviews with patients, psychiatrists working in the on-call system, and nurses at the three regional psychiatric centres that had participated in at least one VC consultation in a psychiatric emergency. This article is part of a larger study [13] that explores how this tele-psychiatric on-call system affords clinical and organisational changes within emergency psychiatry.

### Participants

Twenty-nine participants were recruited for interviews: 5 patients (1 male and 4 females, ages 18 to 51), 5 psychiatrists (5 males, ages 40 to 60) and 19 nurses at the regional centres (4 males and 15 females, ages 39 to 63). All the psychiatrists that participated in the on-call system were interviewed except for the psychiatrist heading the department and who also initiated the new service. Both patients and the nurses represented all the three regional psychiatric centres that participated in the VC consultations. Six of the nurses were also clinical managers for each of the three local psychiatric wards and the three ambulatory psychiatric teams. At the time of their VC consultation, all patients were in an emergency situation during which they were considered for hospital admission. With consideration for their states of health, they were invited to participate in the study when they were discharged from emergency care. All potential participants were invited to participate in the study; i.e., all psychiatrists involved in the on-call system, all nurses at the regional centres that had tried VC and all patients who took part in a video consultation.

### Data generation and analysis

The interviews were all conducted individually between July 2012 and June 2013, which was approximately 1 to 2 years after the VC system became operational. The psychiatrists and the nurses were interviewed at their places of work. Four patient interviews took place at the closest regional psychiatric centre to their home, while one patient was interviewed at her home. The interviews focused on the participants’ experience of using VC consultations to provide psychiatric emergency care. Interview guides were used to structure the dialogue, while at the same time allowing the participants to rethink and explore their experiences in detail through ‘grand tour’ questions [14]. Because patients and service providers were expected to have quite different positions towards the application of VC, three different interview guides were created, each of which was tailored to the patients, nurses or psychiatrists. All participants were asked about their backgrounds, expectations and experiences with the on-call system and the VC consultations. The psychiatrists and nurses were also questioned about clinical, professional and organisational issues, while the patients were asked to explore in retrospect their experience of taking part in a VC consultation in a vulnerable situation. Based on the different interview guides, the interviews with the patients lasted 40 to 50 minutes, while the interviews with the psychiatrists lasted between 65 and 80 minutes. Because of variation in their experiences of using VC, the interviews with the nurses varied in length between 34 and 75 minutes. The interviews were all digitally recorded and then transcribed.

Our analysis followed a stepwise-deductive-inductive (SDI) approach [15] directed towards identifying issues and themes inductively and by ‘emergence’ [16]. The transcripts were first coded in detail with the support of the HyperRESEARCH analysis software, maintaining in very detail the content of interviews. A total of 241 empirically close codes were generated in this process. These codes were grouped into 9 categories and then into 5 main themes. One of these five themes was titled ‘Confidence’ and forms the empirical-analytical basis for this article. Using the stepwise-deductive’ control questions in the SDI method, each following step was assessed. For example, coding names were questioned for each code (e.g., Does it represents in detail what is being said in the interview?). After categories were developed inductively, we asked whether the categories were strong enough to cover the content of the categorised codes. By using this circular strategy, the SDI approach secured a tight connection between the empirical data and our interpretation [15].

### Ethical considerations

The study has been approved by the Regional Committee for Medical Research Ethics in Norway. All participants were recruited based on voluntary participation, all patients provided written informed consent and all health personnel provided oral consent. The data material was depersonalised and securely handled according to the ethical recommendations of the Regional Committee for Medical Research Ethics in Norway.

## Results

The regional psychiatric centres were found to largely operate autonomously. On-call psychiatrists were regularly contacted via telephone by the nurses, although not on a daily basis. Because the use of the telephone was a well-established practice between the regional centres and the psychiatrists, the VC system was mostly seen as a supplement to the existing telephone communication. VC was used in the more challenging situations, in which the nurses or psychiatrists were uncertain about the patient’s assessment and/or further treatment. In most cases, telephone calls were used as dyadic communication; e.g., between one nurse at a regional psychiatric centre and the on-call psychiatrist. When using VC, however, the patient and often a second nurse were also included in the communication.

In the analysis of the participants’ experience with applying VC, various accounts relating to confidence emerged and were grouped into a core empirical theme. We found that having access to the VC system increased the experience of confidence in challenging psychiatric emergencies in four different ways: (1) by strengthening patient involvement during psychiatric specialist assessments, (2) by reducing uncertainty, (3) by sharing responsibility for decisions and (4) by functioning as a safety net even when VC was not used. We explore these accounts in more detail in the following subsections.

### Patient involvement

VC communication between the regional centres and the on-call psychiatrist involved the patient directly, as opposed to the established use of telephones, and strengthened patient involvement. Although some of the patients said they had been sceptical about communicating through VC in advance, they experienced stronger confidence and a feeling of being taken more seriously afterwards the VC consultation. The patients emphasised that the direct contact with the psychiatrist made them calmer and ensured that the right assessment and decisions were being made.

*VC gave me the possibility to speak directly with the psychiatrist while the nurse was sitting next to me. I could actively take part in the assessment.* (Patient 5)

*I may have become annoyed, or maybe offended, if the communication about my mental health had been based on the view of the nurse. It was nice to be able to present my own story [directly to the psychiatrist].* (Patient 3)

This statement by Patient 3 emphasises the need or wish for expert assessment from a specialist and not ‘based on the view of the nurse’. For patients in this situation, experiencing that extra efforts are being made to ensure the highest quality of care is very meaningful. Patient 3 also said, ‘*VC was helpful and calming. I understood that they took me seriously when they had to use VC’.* From this patient’s perspective, the use of VC meant that all available resources were being used to provide the best help possible.

The nurses and psychiatrists also found that VC enabled stronger patient involvement.

*A telephone call is quicker. In spite of that, I am more satisfied with my work after VC because the patient has received a better service.* (Nurse 4)

*Through VC I can see and talk directly with the patient, and then I get a better overview of the situation when I feel it is necessary. A VC consultation provides more than a telephone call.* (Psychiatrist 5)

*When the psychiatrist meets the patient [..] more people have observed and evaluated [the patient], we all know each other’s thoughts, and of course this improves the confidence both for the patient and for us.* (Nurse 5)

The patients got more involved in the communication between the nurse and the psychiatrist when VC was used, and this changed the social dynamics of the consultation. When the voice of the patient was no longer mediated through the nurse, the nurse was relieved of a mediator’s responsibility, as pointed out above. The patient felt that he or she was taken more seriously by being allowed to take part in direct dialogue with the highest level of formal expertise.

### Reducing uncertainty

In challenging psychiatric emergencies, the nurses at the regional psychiatric centres were sometimes uncertain about how to respond; they would then ask the on-call psychiatrist to see the patient. In these situations, they would also want to discuss the patient’s situation and have access to help to collaboratively decide on the best treatment options. In these situations, using VC reduced uncertainty and ambivalence. One of the nurses said that using VC provided an opportunity ‘*to assess the patient together with someone who also can see the patient, who can see the same things as I, or maybe something I haven’t seen, which makes the assessment sufficient’* (Nurse 10). When the psychiatrist had seen the patient, the nurse also gained professional support for his or her preliminary assessment, which improved their confidence in their own skills:

*What I consider most important is that the psychiatrist can watch the patient and assess the patient based on what he sees. This is a support for me, if I have observed the same things.* (Nurse 9)

In challenging situations or when in doubt, the psychiatrists also wanted to see and talk with the patient in order to make well-considered decisions. When VC was not used, a telephone call was made by the nurse to the psychiatrist to present their view and interpretations and to discuss next steps. However, aspects of the patient’s condition that were difficult to convey verbally and that potentially would require direct observation were potentially lost or overlooked when the psychiatrists only received information through the nurse. Therefore, the quality of assessment and decision-making was strengthened through the VC contact between psychiatrist and patient. The psychiatrists said that having the patient visible on the screen in front of them was important.

*I feel more confident when I in fact have seen the patient face to face.* (Psychiatrist 2)

*I am more certain with a decision I might not make otherwise without having seen the patient. [Seeing the patient through VC] is something different than receiving a story told by others.* (Psychiatrist 1)

*At times, when I don’t know the nurse or the patient, and if it is a serious situation, I may feel uncertain. If the story told by the nurse is not in lined with the story from the referring general practitioner, my gut feeling tells me something is not right. Then I may have a need to enter the situation myself.* (Psychiatrist 5)

*I think we have to see the patients. If we cannot meet them face-to-face, we should see them on VC. This service would have been less certain without it, and we might need to take chances. I believe the risk would have been greater if we didn’t meet the patients ourselves with VC* (Psychiatrist 3)

The use of VC was therefore regarded as a method to ensure high-quality decision making, which increased the confidence for all involved that the best decision was being made for each patient. This included the opportunity of receiving a second opinion from a psychiatrist or having immediate access to the experience and competence of a psychiatrist in a challenging situation. Also, during VC sessions, several participants are able to present their views, thereby increasing both the understanding as a group comprised of the clinicians and the patient and the collective confidence in—and commitment to—the decisions being made.

### Sharing responsibility

The use of VC increased the confidence of both the nurses and psychiatrists because more than one person was able to observe and communicate with the patient. VC made it easier to share the responsibility for patient treatment, which was especially important in challenging situations, such as for suicidal patients. For the nurses at the regional centres, sharing the responsibility for decision-making with a psychiatrist was of great support.

*My assessments are supported in situations in which I would otherwise feel alone.* (Nurse 2)

*Assessment of suicidal patients [..] is a responsibility I do not want to have.* (Nurse 8)

*For me, this is all about shared responsibility. [..] VC gives me the opportunity to discuss my concerns, the patient may participate in the discussion, and then decisions can be made.* (Nurse 8)

The psychiatrists emphasised that the nurses at the regional centres in general had made safe and sound patient assessments before contacting the psychiatrists. In some cases, however, the nurses needed a psychiatrist’s confirmation of their assessment. One of the nurses said, *‘It is also important to receive confirmations that you have done and said the right things’.* (Nurse 10)

Although the use of VC was usually initiated by the nurses at the regional centres, some of the psychiatrists reported that they asked for VC when, during a telephone call, they heard that the nurse felt insecure. As Psychiatrist 2 said, *‘I suggest VC when the regional staffs are uncertain and I am unable to form a picture of the patient and the problem’.* The combination of not being able to see the patient with their own eyes and feeling that the nurse was uncertain about, for instance, the seriousness of a situation, was a motivation for initiating VC. This led to the professional support from the on-call psychiatrists being more specific and sensitive to each patient’s case. The psychiatrists’ direct view of the patients provided a stronger experience of a shared responsibility. They became involved as an observer together with the nurse and were not just an external advisor.

### A safety net

Finally, the nurses and the psychiatrists emphasised the availability of VC as a safety net. Although they did not actually use VC very often, the on-call system implied that the psychiatrists could be accessed by the regional centres through VC 24 hours a day. With a specialist only a VC connection away, the healthcare providers felt less uncertain in seriously and challenging psychiatric emergency situations.

*I feel much more confident. [..] I come to work with a much greater degree of calmness than before.* (Nurse 10)

*There is always someone there. [..] I am never alone.* (Nurse 8)

VC as a safety net was reported as being particularly important during the evenings, nights and weekends, when there were fewer health personnel at the centres.

*I often work night shifts, and I know there is always a psychiatrist on call to help me in the event of an emergency. [..] Yes, I do feel it has eased my work.* (Nurse 7)

*I feel more confident when I know we have the VC opportunity. The staffs know it, and the patients know it to some degree. I believe it has a positive contagious effect on us all.* (Psychiatrist 5)

The improved confidence among the nurses may have actually led to more seriously ill patients being admitted to the regional centres rather than sending them to the acute hospital ward in Tromsø. Based on VC as a 24-hour safety net, the nurses knew that they could consult a psychiatrist for further assessment at any time. If the patient’s condition changed, the patient could be reassessed, and decisions could be quickly revised if required.

*The availability of VC is almost as important as the actual [use of] VC. It gives us all a degree of confidence. We can just use it if we need it.* (Psychiatrist 5)

The safety net argument is of major importance in emergency care, as this work is characterised by having to immediately solve problems using any tools at hand. The opportunity of expanding the telephone communication between the psychiatrists and the regional centres by VC has value as an option itself.

## Discussion

In this discussion, we reflect on our findings in a larger context, both theoretically and in relation to practical implications. It is important to note that the use of VC analysed in this paper is within the context of medical emergencies. In both psychiatric and somatic medicine, decisions need to be made within a short timeframe, during which confidence in actors, their handling of tasks and their decision-making is of great importance to effectively address any acute situation. It has been demonstrated that communication technologies that are well-integrated into team collaboration increase quality by applying knowledge and experience across personnel and locations [17]. In previous trials of VC within the context of simulated cardiac arrest, visual contact through VC improved the confidence of both the rescuers and the nurses [18,19]. In addition, hospital teams worked together more efficiently and with greater confidence when VC was used as a collaboration tool for complex medical emergencies and traumas [20,21]. Our study has shown that VC may increase certainty, improve the sharing of information and contribute positively to teamwork in challenging psychiatric situations. The presence of VC as a treatment tool therefore supports preparedness for medical emergencies.

At a more conceptual level, we have identified two main mechanisms for generating stronger confidence within the provision of emergency psychiatric care: (1) using VC for collaborative problem-solving across locations and professional levels that involve patients and (2) maintaining VC as a feasible resource for challenging situations. Both the psychiatrists and the nurses emphasised how the use of VC for collaborative problem-solving across locations and professional levels increased their confidence. By solving a problem through a team process, their uncertainty about the right assessment was reduced, and they shared the responsibility for decisions made in challenging situations.

In our study, to determine if the psychiatrist needed to be contacted, the nurses always performed an initial assessment for each patient. Often the psychiatrists confirmed these assessments; hence, collaborative problem-solving improved the nurses’ confidence in their own skills and competence. This finding is in line with previous studies comparing the effectiveness of VC to telephone calls, which found that the VC groups had increased confidence among participants and improved their ability to collaborate as teams [18-22]. Including the patient in the VC-mediated team process allowed him or her to take an active part in their assessment and felt included in the decision-making process. Patient involvement meant that the patient’s voice could be heard without first being filtered through the nurse.

The second main mechanism that we identified was maintaining VC as a safety net for challenging situations. This safety argument is of major interest because it challenges the idea of frequent use as a success criterion for technological innovations. Having the opportunity to use VC in a difficult situation is a source of confidence even when it is not used. Accordingly, it has been demonstrated that having a specialist’s e-mail address is important for chronically ill patients even if no e-mails are ever sent [23]. For the nurses at the regional psychiatric centres, just knowing that they could access the on-call psychiatrist through telephone or VC whenever they need it made them calmer and feel less alone. This opportunity is also an important aspect of confidence for the psychiatrist; i.e., knowing he can see and talk with the patient directly if he consider that it is necessary. VC as a safety net improved confidence among the nurses. Having the VC system set-up and being trained in how to use it strengthened the regional centres’ ability to handle and admit challenging and more seriously ill patients. A very important finding is that this effect was not dependent on the actual use (or frequency of use) of the VC system; rather, it was only related to having the VC equipment installed and being confident in using it. In addition, all parties knew that the nurses could contact the psychiatrist again later for reassessment if the patient’s situation worsened.

The introduction of VC is a minor technological innovation, as VC technology has been well-established for many years. However, our study confirms that in psychiatric emergencies, including VC into a network between regional psychiatric centres and specialists at a central hospital may trigger significant organisational development. The nurses experienced the support of specialists, both as confirmation of their own assessment and as a safety net for dealing with challenging situations. At the same time, the patients experienced that the VC connection represented a major effort being made for their sake: It was a reassurance of the health personnel taking the needs and worries of patients seriously. Our analysis supports the sociological notion of a paternalistic public health service [24], in which patients trust doctors’ advice more than other health personnel’s assessment. Also, to some degree, this explains the effect of direct contact with the psychiatrist: Being taken seriously is related to the progress of the actual consultation, as well as to the formal position of the clinician who manages the consultation.

The VC installation afforded another level of regional collaboration for handling psychiatric emergencies. By applying an *affordance perspective* [25,26], we emphasise how various actors perceive the application of VC differently and how these different perceptions contribute to organisational changes within the delivery of emergency psychiatric services over time. By having direct access to the psychiatrist, the patients felt that they were taken more seriously. The nurses experienced VC as providing shared responsibility and as a safety net, and the psychiatrists were able to provide better assessments as the patients were visible and given a more active role. While these experiences reflect affordances of VC in psychiatric emergencies, the four themes in our analysis are related to various aspects of confidence that are not related to purely technical qualities. Rather, the potential use of VC provides a greater transparency between the central and local healthcare providers, as well as between these providers and the patients. This may represent a ‘good circle’ of developing greater trust between actors in the provision of emergency psychiatric care.

Our analysis also supports a more reserved belief in the collaboration/education argument for implementing tele-medical applications: Stronger professional confidence among local healthcare staff may in the long run give them a more independent position in relation to centrally placed specialists. However, due to the inclusion of patients’ voices in our analysis, we have identified that patients in very vulnerable situations have a need for direct access to the highest level of formal expertise. Although well-founded assessments were made by the nurses in this study, patients submit to a taken-for-granted warranty in a doctor’s clinical gaze. While health service provision is becoming more interdisciplinary, specialised health advice today comes from many more sources than just medical doctors. However, physicians’ knowledge and practice hold a strong position in the general population [24], and this needs to be taken into consideration when handling particularly vulnerable mentally ill patients.

### Limitations

This study is limited by the fact that this new VC system had only been in use for 1 to 2 years before the interviews were conducted. The patients and most of the nurses had therefore participated in only one VC session, although the psychiatrists had used VC several times. The participants may have focused on other aspects of their experience if the system had been well- established or if they had more experience with VC in this setting.

The study is also limited by the few patients willing to participate. Even if the total number of interviews were sufficient for understanding how VC is experienced and used, more patients could have helped us to better understand the patient perspective. We cannot rule out the fact that the patients willing to participate were more positive to VC than those who did not reply positively to recruitment.

Using semi-structured interviews enabled us to explore the nuances of the experiences of both the providers and patients in VC consultations. While the accounts of participants in this study are not statistically generalisable, the analysis in this article can serve as a basis for a conceptual [15] or analytic [27] generalisation, in which the four processes of confidence are relevant to other uses of VC in medical emergencies. Therefore, the contribution of this article is first and foremost an empirical exploration focused on this conceptual understanding of confidence.

## Conclusion

This study has demonstrated that an emergency psychiatric service delivered by VC may improve the confidence of psychiatrists, nurses and patients in challenging psychiatric emergencies. Even if VC is not used often, it serves as a safety net for challenging situations, which increases the confidence of both nurses and psychiatrists. This illustrates that frequent use is not necessarily a success criterion for the application of technological innovations to emergency care.

When VC is used as an alternative to telephone calls, a group of people can see and hear each other at the same time. This means that all participants—patients, nurses and psychiatrists—can take an active role in the assessment and decisions-making process through face-to-face communication. VC is a richer mean of communication through which participants, especially patients, experience increased trust in each other and improved confidence in the decisions that are made. We therefore conclude that VC can be an important tool for building confidence in psychiatric emergencies and that patients, nurses and psychiatrists experienced that VC enables the provision of high-quality services for patients who need emergency psychiatric care that is located close to their homes.
